# Carbon nanodot with highly localized excitonic emission for efficient luminescent solar concentrator

**DOI:** 10.1515/nanoph-2023-0578

**Published:** 2023-10-10

**Authors:** Jinhao Zang, Fuhang Jiao, Jianyong Wei, Qing Lou, Guangsong Zheng, Chenglong Shen, Yuan Deng, Ehsan Soheyli, Reza Sahraei, Xun Yang, Huaping Zang, Weimin Zhou, Wei Fan, Shaoyi Wang, Lin Dong, Chong-Xin Shan

**Affiliations:** Henan Key Laboratory of Diamond Optoelectronic Materials and Devices, Key Laboratory of Materials Physics, Ministry of Education, School of Physics and Microelectronics, Zhengzhou University, Zhengzhou, China; State Key Laboratory of Advanced Optical Communication Systems and Networks, University of Michigan – Shanghai Jiao Tong University Joint Institute, Shanghai Jiao Tong University, Shanghai, China; Department of Physics, Faculty of Science, Ilam University, Ilam, Iran; Department of Chemistry, Faculty of Science, Ilam University, Ilam, Iran; Science and Technology on Plasma Physics Laboratory, Laser Fusion Research Center, China Academy of Engineering Physics, Mianyang, China

**Keywords:** carbon nanodot, localized exciton, luminescent solar concentrator, photovoltaics, waveguide

## Abstract

Luminescent solar concentrators (LSCs) are attractive for the easy operation and high compatibility with building integrated photovoltaics due to their low cost, large-scale and applicability. However, underutilized sunlight in visible wavelengths often impedes the advance of LSCs. Here, we demonstrate an orange-emitting carbon nanodots-based LSC (O-CDs) with excitation concentrated in the visible wavelengths. The orange-emitting carbon nanodots (O-CDs) with highly localized excitonic emission are prepared via atomic condensation of doped pyrrolic nitrogen, delivering a high photoluminescence quantum yield of 80 % and a suitable Stokes shift with absorption spectrum situated in the visible region. The O-CDs are embedded in polyvinylpyrrolidone to obtain a highly transparent, stable and environmentally friendly O-CDs-based LSC. Thanks to efficient utilization of solar radiation in visible areas and well match between the emission of O-CDs and the response bands of photovoltaic cells, the O-CDs-based LSC reveals an optical conversion efficiency of 5.17 %, superior to that of most carbon nanodots-based LSCs. These results provide an effective strategy to develop carbon-based luminescent concentrated materials for architectural integrated photovoltaic technology.

## 1 Introduction

Solar energy as an environmentally friendly and renewable energy has attracted a great deal of attention, accelerating the continuous progress in eco-friendly photovoltaic (PV) technologies. However, it is still difficult to achieve the desired goal of so-called near zero energy buildings (NZEBs) in a highly urbanized environment, due to the lack of rooftop space in tall buildings and the high cost of land for ground PV installation [1, 2]. One promising solution is to integrate the cost-effective luminescent solar concentrators (LSCs) into semi-transparent glazing systems to collect the sunlight from the facades of urban buildings for PV generation. Meanwhile, the adoption of LSCs as “invisible” architectural integrations can well take into account architectural aesthetics, unlike other opaque or electrode-based semi-transparent PV modules [3–5]. In addition, as a novel and efficient solar energy collector, LSCs possess significant advantages and versatility in terms of shape, size, transparency, flexibility, and cost, this renders them highly promising for diverse applications in the fields of building integration photovoltaics, displays, garden landscaping, photothermal sensing, and light re-sources for electrocatalysis. Consequently, they are poised to play a pivotal role in the next generation of eco-friendly photovoltaic technology [6–9].

To realize cost-effective photovoltaic energy harvesting, luminescent solar concentrators (LSCs) are usually adopted to collect more solar light with less expensive materials. Typically, LSCs are constructed by utilizing transparent glass or polymer as the optical waveguide, which is coated or embedded with fluorophores such as organic dyes and nanocrystal quantum dots. The fluorophores absorb solar photons and emit long-wavelength light, which can be concentrated to the edge of the LSC through total internal reflection and collected by the PV cells for power generation. For this purpose, numerous fluorophores such as organic dyes or organic–inorganic hybrid chromophores have been proposed to achieve high efficiency and practical LSC devices. However, such fluorophores are prone to suffer from poor photostability, undesired spectral tunability and relatively large overlap between the absorption and emission spectra, leading to unsatisfactory performance of LSCs [10–13]. Nanocrystal quantum dots (QDs) seem to be a solution to fabricate efficient LSCs on account of their broad tunable absorption/emission spectra across the visible and near-infrared (NIR) wavelengths, near-unity photoluminescence quantum yield (PL QY), superior chemical/photostability to organic dyes or hybrid materials [3, 14]. Nevertheless, unacceptable toxicity caused by the heavy metal components (Cd, Pb) and relatively high cost of quantum dots limit the development of LSCs based on quantum dots. Although some quantum dots, such as Mn or Cu-doped QDs, silicon QDs, are not plagued by toxicity, the complex synthesis process hinders the large-scale and commercial application of QDs-based LSCs [15, 16]. Carbon nanodots, as a new type of fluorescent nanomaterial, show unique physical and chemical properties. So far, multi-color preparation of carbon dots in the visible range has been achieved, and its fluorescence quantum efficiency is constantly updated, and its good biocompatibility, tunable luminous wavelength and other characteristics make it have been widely used in biological detection, fluorescence imaging, information encryption other aspects [17–21]. In addition, due to its low cost, simple synthesis, rich functional groups and high stability, it is also considered to be a promising candidate for LSCs emitter materials [22–28]. In addition to emitters with high PL QY, large Stokes shift and long-wavelength emission matching PV devices are commonly expected in the fabrication of LSCs [13, 29–34]. Nevertheless, absorption by emitters with large Stokes shifts and long-wavelength emission matching PV devices is typically concentrated in the ultraviolet band, whereas the solar radiation is more concentrated at visible wavelengths. Thus, most of the reported LSCs with CDs as emitter suffer from problems such as weak solar radiation capture ability, low light–light conversion efficiency and undesirable light/thermal stability. Hence, it remains crucial to develop CDs with high PL QY, appropriate Stokes shift with absorption at visible wavelengths and emission matching PV devices for the development of LSC technologies that fully and efficiently utilize solar energy.

**Figure 1:**
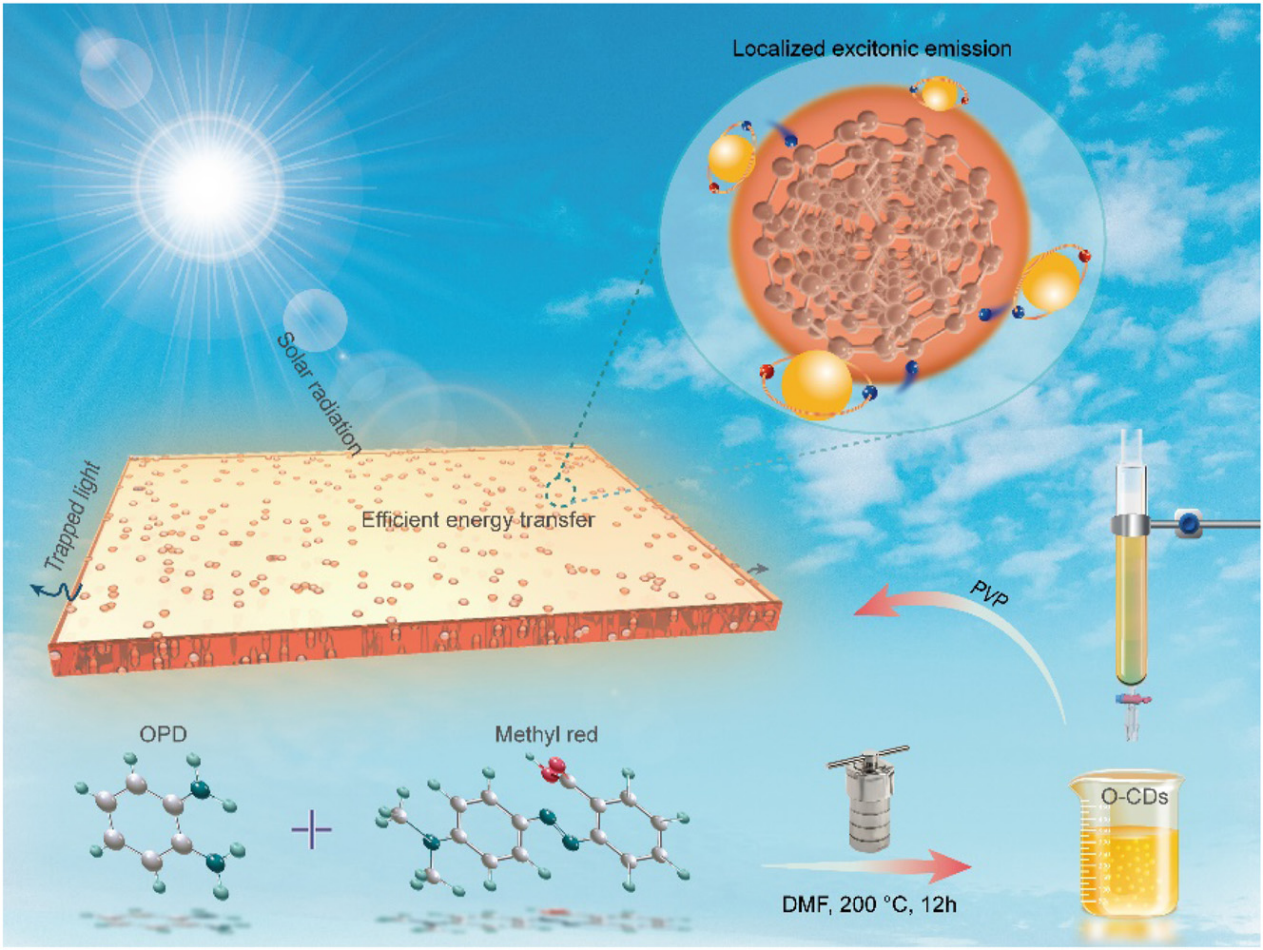
Design principles of efficient LSCs based on O-CDs with localized excitonic luminescence.

Here, we present a LSC based on orange-emitting CDs (O-CDs) with highly localized exitonic emission (Figure 1). The O-CDs served as emitters of LSCs are simple prepared via a solvothermal method yet deliver a high PL QY of 80 %. The absorption of O-CDs concentrated at visible wavelengths and the emission at 575 nm that well matched with silicon-based PV devices enable effective solar radiation capture rate and high performance of O-CDs-based LSC. By embedding O-CDs into polyvinylpyrrolidone (PVP) and coating on standard commercial glass, the LSC with well biocompatibility, high transparent and large Stokes shift of 0.44 eV situated in the visible wavelengths is developed. The obtained O-CDs-based LSC achieves a relatively high optical conversion efficiency of 5.17 %, outperforming previous pure CDs-based LSCs. In addition, the O-CDs LSC exhibits excellent photo- and air-stability without any significant variability even after three months of exposure to natural light, demonstrating great promise in photovoltaic energy harvesting.

## 2 Experimental section

### 2.1 Materials

Methyl red (AR, Aladdin), *o*-phenylenediamine (98 %, Aladdin), and polyvinylpyrrolidone (PVP) (Aladdin) were used as precursors to prepare O-CDs and LSC films. N,N-dimethylformamide (DMF) (99.5 %, Aladdin) was used as reaction solvent. All chemicals were used as received without further purification.

### 2.2 Synthesis of O-CDs

The CDs were prepared by solvothermal method modified from our previous reports [35, 36]. Methyl red (0.1 g) and *o*-phenylenediamine (0.1 g) were dissolved in 10 ml DMF. Next, the mixture was transferred into a Teflon-lined stainless-steel autoclave (25 ml) and heated at 200 °C for 12 h. After the reaction, the reactor was cooled down to room temperature. Then the as-synthesized CDs were purified via silica column chromatography using dichloromethane as the eluent and finally dispersed in dichloromethane (CH_2_Cl_2_) for further characterizations.

### 2.3 LSCs based on O-CDs

First, the O-CDs were added into the polymer matrix to obtain the precursor. 20 mg O-CDs were dispersed in 10 ml CH_2_Cl_2_ and then mixed with 8 g PVP (0.25 %) under stirring until the PVP was completely dissolved. Next, a 10-min ultrasonic treatment was performed to completely remove the bubbles in the mixture to obtain the homogeneous ropy O-CDs/polymer. Then, the mixture was dropped on a commercial glass substrate and smoothed by knife coating using a blade (BEVS 1806B). Finally, the O-CDs-based LSC was obtained by evaporating CH_2_Cl_2_, in which a homogeneous and transparent film was formed over the substrate.

### 2.4 Characterization method

Transmission electron microscopy (TEM, Thermo Scientific Talos F200S, USA) was used to obtain high-resolution TEM images of O-CDs. The X-ray diffraction (XRD, Bruker D8 Discover, Germany) spectra were recorded to analyze the crystal structure of O-CDs. The X-ray photoelectron energy spectra (XPS, Kratos AXIS HIS 165, UK) were collected to analyze the composition of O-CDs. Fourier transform infrared (FTIR, Thermo Scientific Nicolet IS10, USA) spectroscopy was measured to confirm the functional groups of O-CDs. The PL spectra of O-CDs solution and the LSCs were collected using a fluorescence spectrophotometer (Hitachi F-7000, Japan). The UV–vis absorption and transmittance spectra were recorded by an ultraviolet-visible spectrophotometer (Hitachi UH4150, Japan). Fluorescence lifetimes were measured by a spectrophotometer (Horiba Fluorolog-3 FL3-22, France) using a 405 nm. A solar simulator (Pharos Technology PT-IVM3S+, USA) at AM 1.5G (100 mW/cm^2^) and commercial monocrystalline Si solar cells were used to study the external optical efficiency of the O-CDs-based LSCs.

## 3 Results and discussion

**Figure 2:**
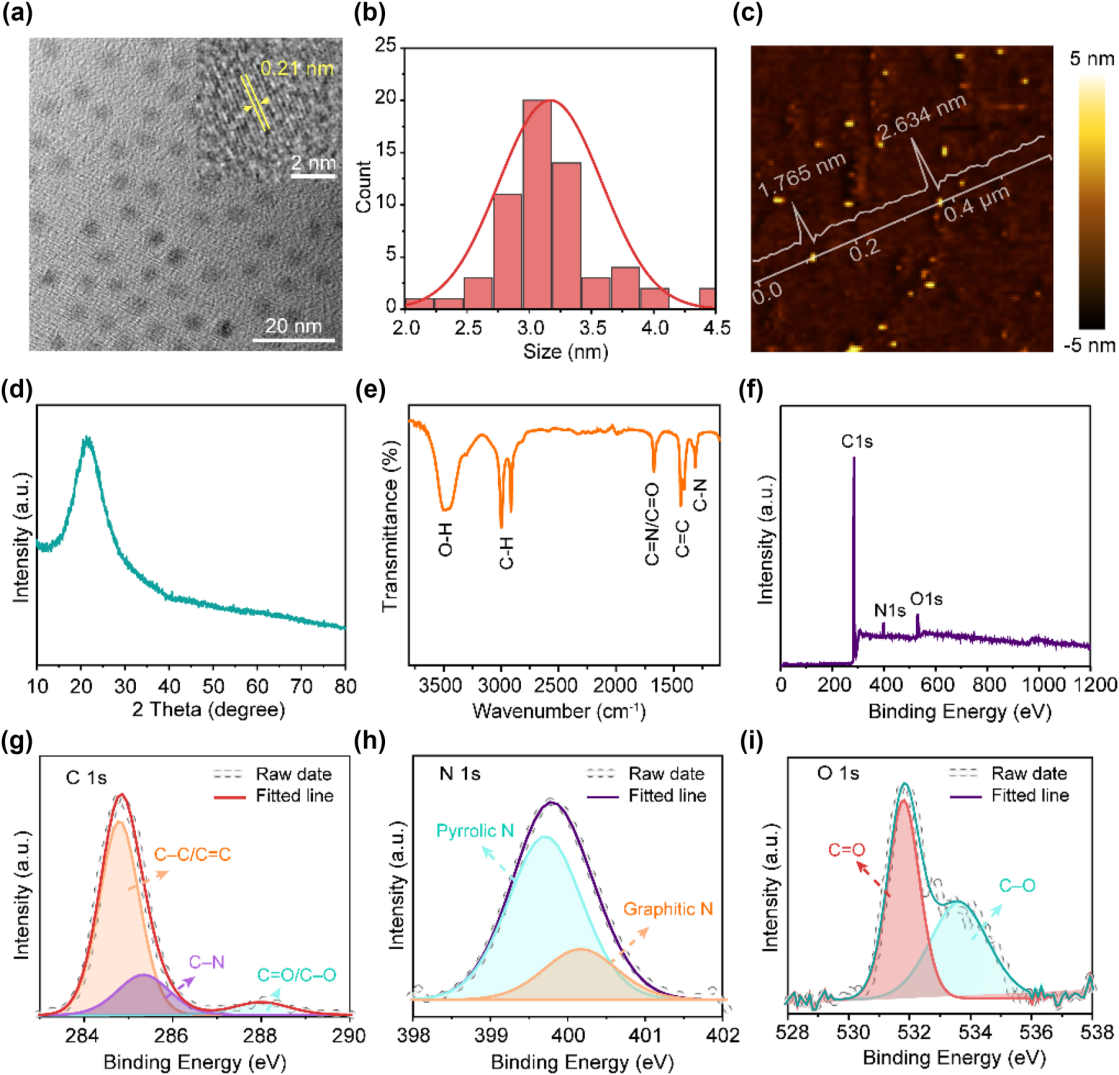
Morphology and structural characterization of the O-CDs. (a) TEM image of the O-CDs with HRTEM image in the inset. (b) Size distribution of O-CDs. (c) AFM image of the O-CDs. (d) XRD pattern of the O-CDs. (e) FT-IR spectra of the O-CDs. (f) XPS spectra of the O-CDs. High-resolution XPS spectra of the O-CDs, (g) C 1*s*, (h) N 1*s*, and (i) O 1*s*.

As the protagonist of O-CDs-based LSC, O-CDs were prepared using *o*-phenylenediamine (OPD), methyl red, and N,N-dimethylformamide (DMF) as precursors, solvent heat treated at 200 °C for 10 h, followed by silica gel column chromatography. The transmission electron micros-copy (TEM) image (Figure 2a) shows that the obtained O-CDs exhibit a relatively well dispersion and render a uniform particle size of about 3.2 nm (Figure 2b). The high-resolution TEM image of O-CD (inset in Figure 2a) reveals discernible crystalline lattice fringes with a lattice spacing of about 0.21 nm, corresponding to the (100) in-plane lattice spacing of graphitic carbon [37]. According to atomic force microscopy (AFM) result (Figure 2c), the O-CDs with a height of 1.5–3 nm and possess a homogeneous morphology, in agreement with the TEM results. The X-ray diffraction pattern exhibits a broad peak at ≈ 21° (Figure 2d), corresponding to the (002) planes of graphitic carbon, suggesting the *sp* ^2^ hybrid carbon-core structure of O-CDs [38]. In addition to the rigid carbon skeleton core, abundant functional groups exist on the surface of O-CDs. The Fourier transform infrared (FT-IR) spectrum of O-CDs displays characteristic peaks at 1668.7, 1436.3, and 1309.9 cm^−1^, which can be assigned to the stretching vibrations of C=N/C=O, C=C, and C–N, respectively (Figure 2e), indicating the O-CDs have poly-heteroaromatic structures [39]. The peak at around 3494.1 cm^−1^ is attributable to –OH stretching vibrations, demonstrating the existence of –OH bonds on the surface. To further investigate chemical composition information of the O-CDs, X-ray photoelectron spectroscopy spectra (XPS) are performed. Figure 2f show the full survey spectra of O-CDs with three typical peaks, i.e., 284.8 eV (C 1*s*), 398.8 eV (N 1*s*), and 531.8 eV (O 1*s*). The deconvoluted high-resolution C 1*s* spectra of O-CDs consists of three peaks at 284.8 eV, 285.3 eV and 288.0 eV (Figure 2g), corresponding to *sp* ^2^/*sp* ^3^ carbons (C–C/C=C), nitrous carbons (C–N), and carbonyl carbons (C=O/C–O), respectively [40]. The relatively high content of *sp* ^2^ hybrid carbon atoms implies a rigid π-conjugated skeleton structure of O-CDs. Figure 2f demonstrates the deconvoluted high-resolution N 1*s* spectra consists of pyrrolic N (399.7 eV) and graphitic N (400.2 eV), indicating conjugated N groups exist on the surface of the O-CDs [26]. The conjugated N usually acts as localized excitons trapping sites leading to strong emission [35]. The O 1s spectrum contains two components associated with C=O at 531.8 eV and C–O at 533.6 eV (Figure 2i). To sum up, O-CDs exhibit a conjugated aromatic structure and abundant N and O functional groups on the surface.

**Figure 3:**
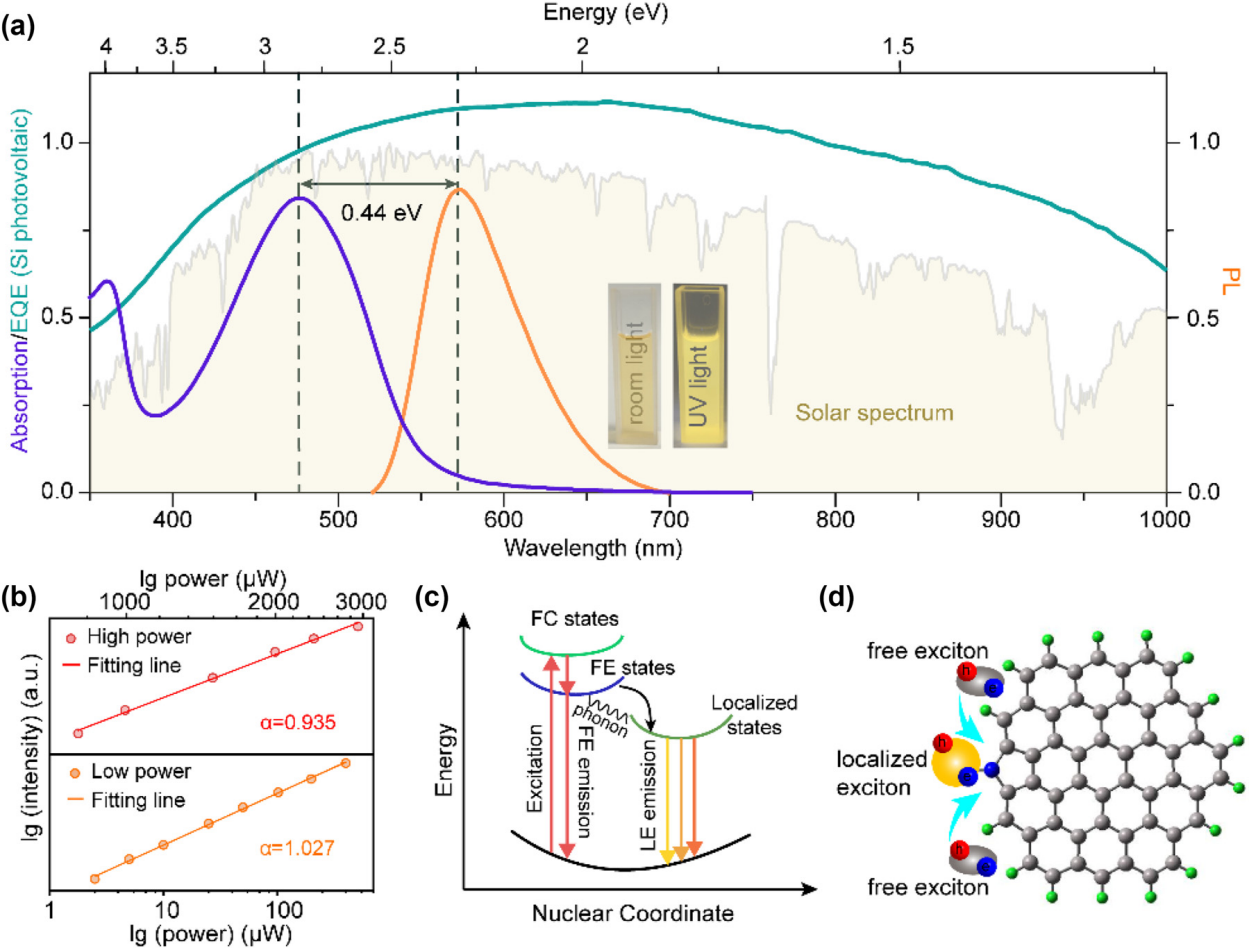
Localized excitonic emission from the O-CDs. (a) Absorption spectra (purple line) and PL spectra (orange line) of the O-CDs in dichloromethane. Photoluminescence was measured at 365 nm. The green line is the EQE curve of the c-Si solar cells we used. The gray shadow shows spectra of solar radiation at sea level. (b) Power-dependent PL spectra of O-CDs. (c) Schematic diagram of the excitonic luminescence in O-CDs. FC states for free carrier; FE states for free exciton and LE states for localized exciton. (d) Schematic diagram of the O-CDs with localized excitonic emission.

The optical absorption and fluorescence spectra of O-CDs in dichloromethane are compared to solar radiation spectrum and a typical external quantum efficiency (EQE) spectrum of the Si photovoltaic cell we used (Figure 3a). The excitation–emission matrix in Figure S1 reveals a stable emission center at around 575 nm as the excitation wavelength ranges from 330 to 600 nm, i.e., the emission of CDs is independent of the excitation wavelength. The absorption spectrum shows two excitonic absorption band peaks at 360 nm and 477 nm, corresponding to the *n* → π* transitions of the C=O and C=N bonds, respectively [26], which is corresponding with the excitation spectra. The O-CDs employed in the LSCs perform a distinct orange emission at a wavelength of 575 nm, which closely matches the EQE spectrum of Si photovoltaic cells, meaning that the emitted photons can be converted into current more efficiently. Comparing the PL spectrum and absorption spectrum, the first excitonic absorption peak centered at 477 nm (2.60 eV) while the PL peaked at 575 nm (2.16 eV), indicating a Stokes shift of 0.44 eV. Note that the absorption of O-CDs is mainly concentrated in the visible band, where the stronger radiation energy appears relative to the ultraviolet region from the solar spectrum, favoring a more efficient use of the solar ray energy. Simultaneously, the small overlap between the excitonic absorption and the PL spectrum induces a reduced self-absorption of the O-CDs, enhancing the fluorescence efficiency. The absolute PL QY of O-CDs was determined to be 80 % at 450 nm excitation using an integrated sphere (Figure S2). The O-CDs can be excited efficiently with barely PL shift over a wide range of excitation wavelengths from 330 to 510 nm (Figure S3), suggesting that the emission of O-CDs is independent of the excitation wavelength [41]. Power-dependent fluorescence spectra were measured to further explore the luminescence mechanism of O-CDs (Figure 3b). The PL intensity of O-CDs tends to sublinear power dependence with the excitation intensity increasing from low power (1 < *P* < 500 μW) to high power (500 μW < *P* < 3000 μW). The value of fluorescence intensity and excitation power (*α*) reduced from 1.02 to 0.93, where *α* values close to 1 indicate single exciton recombination, while *α* values less than 1 indicate local exciton state emission [42]. The saturation behavior of the luminescence in the high-power region suggests that the emission of the O-CDs may originate from the radiative recombination of localized excitonic species. In addition, the PL emission of CDs under different temperature was further characterized (Figure S4), the integrated PL intensity and full width at half maximum (FWHM) at different temperature of the CDs could be fitted with a single exponential decay, indicating the possible PL emission from exciton recombination. With the Arrhenius curve, the value of the exciton binding energy (*E*_b_) and Huang Kun factor (*S*) for the CDs could be fitted (Note S1). The calculated *E*_b_, *S* and *hω*_photon_ were 55.79 meV, 1.64 and 106.22, respectively. Such high *E*_b_ and *S* indicate that the free excitons can be easily captured by the localized state and turned into localized excitons. The above results confirm that the emission of CDs is attributed to the radiative recombination of localized excitonic. The possible origin of the distinct photophysical behavior mediated by the CD exciton species can be described in terms of exciton theory (Figure 3c). After O-CDs is photoexcited, the free excitons produced from the ground state are transported to the nearest emission traps and then transformed into localized bound excitons via strong binding interactions with the photons. The highly localized effect promotes large relaxation energy release, inducing a large Stokes shift of the CD. The O-CDs with localized excitonic emission are schematically depicted in Figure 3d. Bound excitons may facilitate the transition of emissive decay from localized sites and ultimately induce high PL QY for O-CDs.

**Figure 4:**
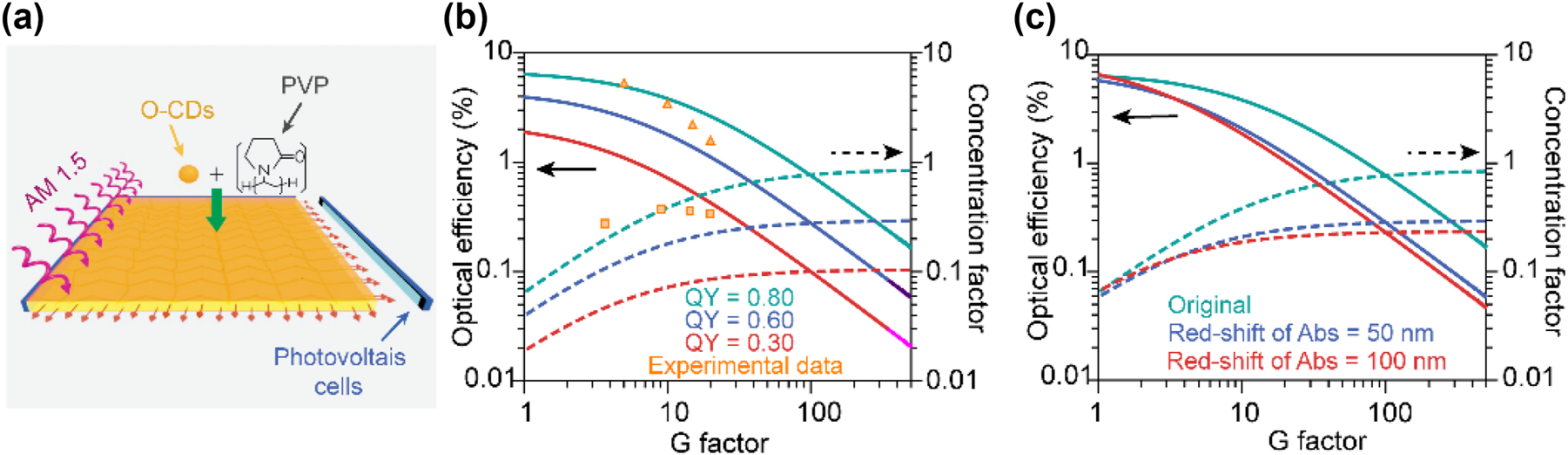
LSCs performance derived from the O-CDs. (a) Schematic illustration of the LSCs. (b) Calculated optical efficiency and concentration factor of the LSCs based O-CDs with PLQY of 0.80 (green lines), 0.60 (blue lines) and 0.30 (red lines) as a function of G factor. The solid and dashed lines indicate optical efficiency and concentration factor, respectively. (c) Calculated optical efficiency and concentration factor of the LSCs based O-CDs with different red-shift of the absorption of the LSCs. The orange triangles and squares indicate experimental optical efficiency and concentration factor, respectively.

**Figure 5:**
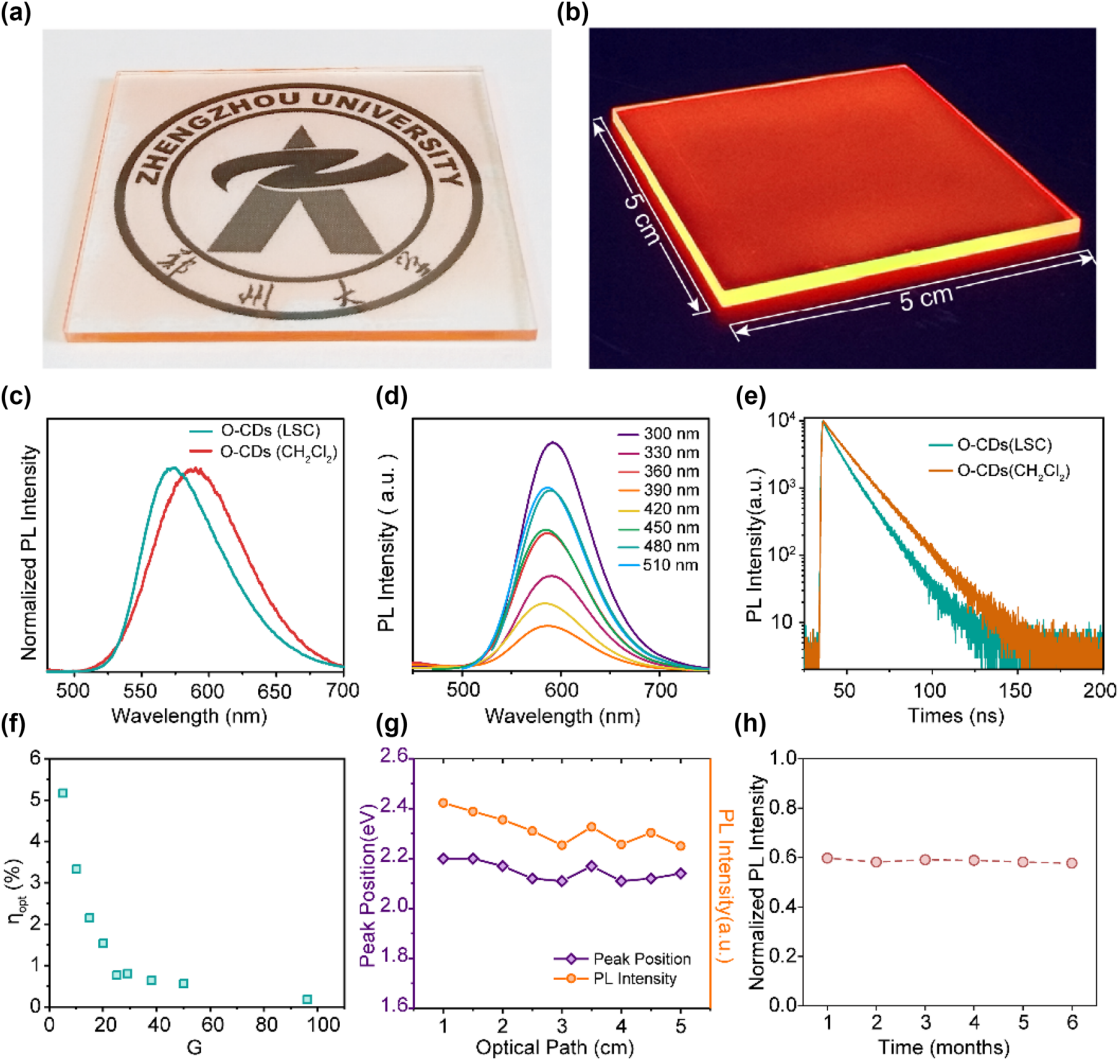
Photophysical properties of the O-CD-based-LSCs. (a) A photograph of a colorless LSC (5 × 5 cm). (b) Photograph of an LSC based on O-CDs illuminated with the UV-light at 365 nm. (c) PL spectra of the O-CDs in LSC and dichloromethane. (d) PL spectra of the LSCs based O-CDs under different excitation wavelengths. (e) PL decay curves collected at emission peak of 590 nm for the LSC based O-CDs and O-CDs solution. (f) External optical efficiency of the LSCs with different *G* factors. (g) PL intensity and emission peak of the LSCs as a function of optical path. (h) PL intensity of the LSCs based O-CDs illuminated by AM 1.5 for 6 months.

O-CDs are a promising LSCs material with excellent optical properties, small self-absorption and long emission wavelength. To reduce the light scattering loss, we fabricated LSCs in a membrane-on-slab structure. Solar-to-electrical PCE measurements were performed using edge-coupled high-efficiency Si solar cells to evaluate the O-CDs LSC performance, schematically depicted in Figure 4a. One crucial parameter of LSCs is the external optical efficiency associated with different geometric (*G*) factors. The *G*-factor is defined as the ratio of the top surface area of the LSCs to the active area of the solar cells attached to the edges of the LSCs. The external optical efficiency (*η*_opt_) can be calculated as:

(1) $$\eta_{\text{opt}}=\frac{l_{\text{LSC}}\times A_{\text{PV}}}{l_{\text{SC}}\times A_{\text{LSC}}}=\frac{l_{\text{LSC}}}{l_{\text{SC}}\times G}$$

where *I*_LSC_ is the short circuit current generated by the solar cell attached to the edge of LSCs, *I*_SC_ is the short circuit current of the same solar cell under direct illumination with the same light condition, *A*_PV_ is the area of the solar cell, and *A*_LSC_ is the area of the top surface of LSCs. We firstly establish a theoretical model of LSCs to investigate the effect of PL QY and Stokes shift on *η*_opt_ of the LSCs (analytical model see Supplementary material). Here we introduced another parameter, concentration factor (*C* = *G* × *η*_opt_), to describe LSC’s ability to collect light [43]. Figure 4b shows the *η*_opt_ and *C* of LSCs based on different PL QY emitters, where the *G* factor increases, *η*_opt_ decreases rapidly while *C* increases. PL QY plays an important role in *η*_opt_ and *C* of LSCs, while limit on regulating the variation trend of *η*_opt_ and *C*. We further explore the influence of Stokes shift on the changing rates of the *η*_opt_ and *C* by shifting the absorption spectrum of the emitters, shown in Figure 4c. When the absorption spectra were redshifted to 50 nm and 100 nm, the Stokes shift decreased by 0.20 eV and 0.40 eV. With the increase of *G* factor, the *η*_opt_ of LSCs decreased faster and the *C* factor increased more slowly, which was consistent with the expectation. Concerning practical applications, the Stokes shift is quite significant since the window glasses of buildings typically have large *G*-factors above 500. Above results reveal that while PL QY of the emitters determines the value of the *η*_opt_ and *C* factors to a large extent, the Stokes shift will greatly affect the changing rate of the *η*_opt_ and *C*. Then, we investigated the performance of O-CDs-based LSC experimentally. Figure 5a exhibits the optical photograph of O-CDs-based LSC with high transparency, where the pattern behind it can be clearly presented. The transmittance of O-CDs-based LSC in the visible spectrum can be maintained at more than 78 %, as shown in Figure S5, which may support its potential application as a window in buildings. When exposed to UV light, the edges of LSCs with dimensions of 5 × 5 cm^2^ glow strongly orange (Figure 5b). After being embedded in PVP polymer matrix, the PL peak centered at 590 nm under 365 nm exhibit a light red-shift compared to the samples in dichloromethane (Figure 5c), which caused by the solvent effect [44]. PL peaks of O-CDs LSC remain constant under excitation at different wavelengths from 300 nm to 510 nm, as shown in Figure 5d. The PL spectrum of O-CDs based on LSC shows a large Stokes shift and less overlap with UV–vis absorption spectrum, which can effectively inhibit self-absorption and reduce energy loss. PL decay curves of O-CDs embedded in LSC and O-CDs in CH_2_Cl_2_ solution are shown in Figure 5e. The PL lifetime of O-CDs embedded in LSC was 9.73 ns, lower than that of O-CDs in CH_2_Cl_2_ solution (13.46 ns). The lifetimes of O-CDs in both environments are on the nanosecond timescale, implying that O-CDs only exhibit fluorescent properties. The PL QY and transmittance of LSC with different CDs concentrations was further explored. It can be found that the PL QY have the best value in the concentration of CDs at 0.25 % (Figure S6), which can be attributed to the aggregation quenching effect of CDs in higher concentration. In addition, the transmittance of LSC decreased with increasing the concentration of CDs, especially in blue region (Figure S7), which is not conducive to the design of architectural aesthetics. To trade off higher PL QY and transmittance, LSC with a CDs concentration of 0.25 % was investigated in following research.

We then investigated the external optical efficiency of the LSCs with different G factors by adjusting the width of the LSCs (1–5 cm) and the size of the solar cells (Figure 5f). When *G* factor is 5 (5 × 5 × 1 cm^2^), *η*_opt_ can reach 5.17 %, which belongs to the high value in CDs-based LSCs (Table S1) [29], [30], [31], [32, 45], [46], [47], [48]. Note that the experimental data are in good agreement with the theoretical simulation, as shown in Figure 4b. However, *η*_opt_ decreases rapidly as the *G* factor increases from 5 to 96, and is 0.19 % when the *G* factor increases to 96, which can be attributed to scattering loss and increased self-reabsorption as the LSCs size increases. To further understand the influence of light transmission path on LSC performance, we measured PL spectra of LSCs with different optical paths using a 365 nm LED as point light source. Different optical paths are achieved by varying the distance of the LED to the side of the LSCs to be tested. As shown in Figure 5g, when the optical path varies from 1 cm to 5 cm, the PL intensity of LSC decreases by about 19 %, which means that a part of the light escapes from the waveguide. In practice, although the sunlight is considered to be parallel light, the larger size of LSC still has a larger optical path in the optical waveguide, which leads to a certain scattering loss. Nevertheless, the PL peaks remains stable at about 2.1 eV (590 nm) which is attributed to the large Stokes shift and the small self-reabsorption, indicating that the emitted light can be stably diffused in the waveguide. Therefore, a higher external optical efficiency can be achieved by selecting an LSC with a suitable optical path to suppress scattering losses.

In order to characterize the stability of LSCs in practical applications, we placed LSCs under the same solar illumination and collected the luminescence intensity under different illumination times. After that, the luminescence intensity and color of the O-CDs LSC remained stable even after 6 months of expose to natural light (Figure 5h and Figure S8), demonstrating the potential of O-CDs LSC for practical applications.

## 4 Conclusions

In summary, O-CDs with highly localized excitonic emission was simple prepared through a solvothermal method and achieved a high PLQY of 80 % and a large Stokes shift of 0.44 eV. By embedding O-CDs into PVP and coating them on commercial glass, we fabricated O-CDs-based LSC in a membrane-on-slab structure that is metal-free, low-toxicity, and cost-effective. O-CDs-based LSC exhibits high transparency and emit a strong orange light in both ultraviolet and natural light. In addition, due to the large stokes shift of O-CDs, LSCs can effectively reduce self-absorption and achieve a high external optical efficiency of 5.17 % (*G* = 5). Moreover, the O-CDs-based LSC remained in stable working condition even after being ex-posed to natural light for 6 months. Therefore, the O-CDs LSC shows promising prospects for solar radiation harvesting.

## Supplementary Material

Supplementary Material Details
