# Supplementary material for: Carbon nanodot with highly localized excitonic emission for efficient luminescent solar concentrator
Source: Nanophotonics. 2023 Oct 10;12(21):4117–26. doi: 10.1515/nanoph-2023-0578 (PMC11501919; doi:10.1515/nanoph-2023-0578)
Supplement: Supplementary file 1 — Supplementary Material Details [file j_nanoph-2023-0578_suppl_001.docx]

Supplementary information

Jinhao Zang, Fuhang Jiao, Jianyong Wei, Qing Lou^*^, Guangsong Zheng, Chenglong Shen, Yuan Deng, Ehsan Soheyli, Reza Sahraei, Xun Yang, Huaping Zang, Weimin Zhou, Wei Fan, Shaoyi Wang, Lin Dong^*^ and Chong-Xin Shan^*^

Carbon nanodot with highly localized excitonic emission for efficient luminescent solar concentrator


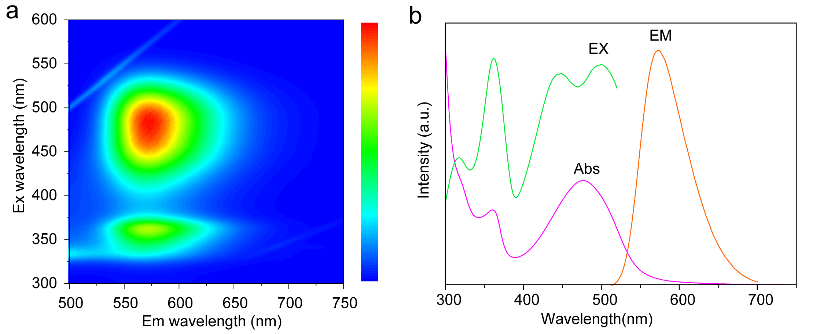


**Fig. S1:** (a) Excitation−emission matrix. (b) the absorption, excitation and emission spectra of CDs.


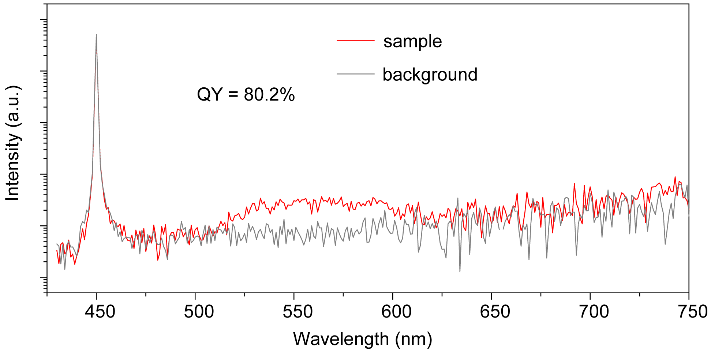


**Fig. S2:** The quantum yield test spectra of CDs with the excitation 450 nm.


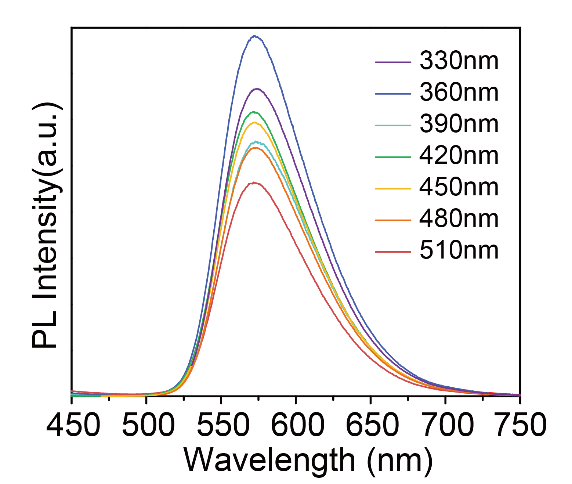


**Fig. S3:** PL spectra of the O-CDs under different excitation wavelengths.


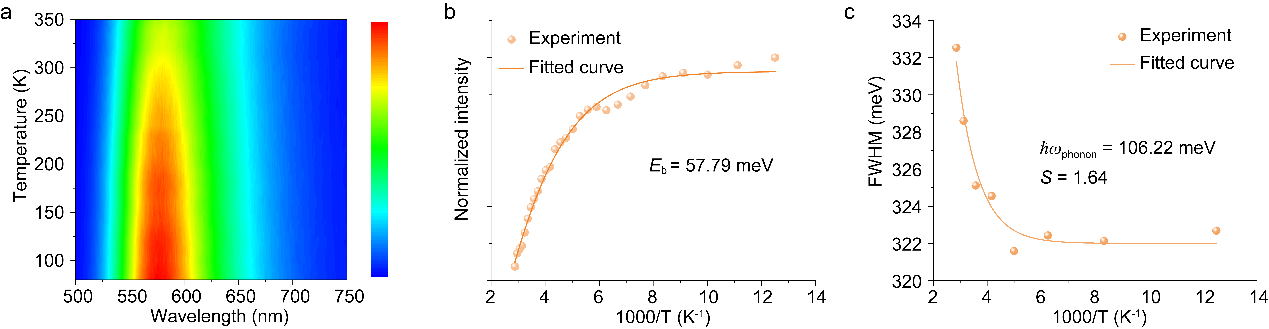


**Fig. S4:** (a) Temperature-dependent PL spectra ranging from 10 to 300 K. (b) Integrated PL intensity and (c) FWHM as a function of reciprocal temperature.

**
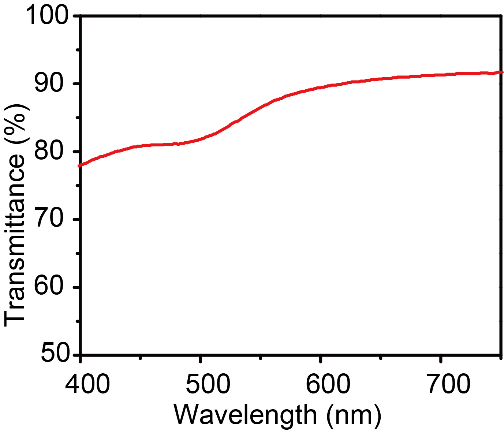
**

**Fig. S5:** Transmittance spectrum of the LSC based O-CDs.


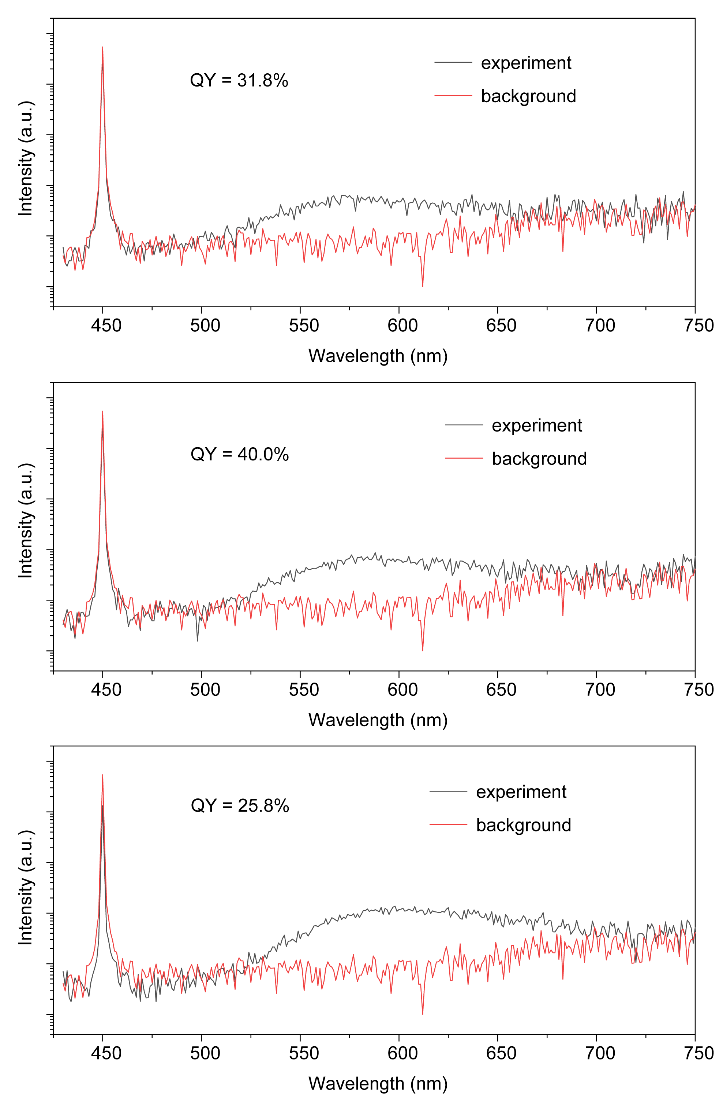


**Fig. S6:** The quantum yield test spectra of different concentrations carbon nanodots based LSC CDs.


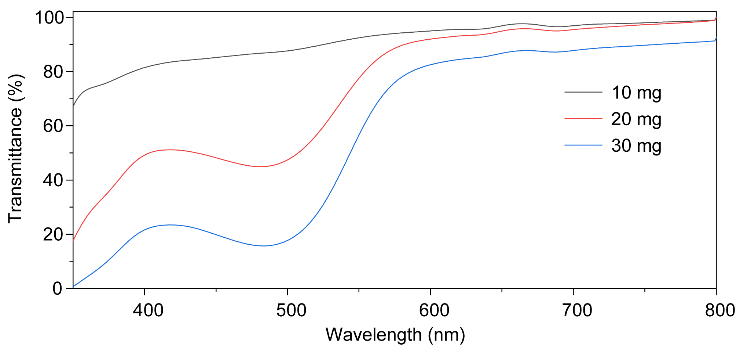


**Fig. S7:** The transmittance spectra of different concentrations carbon nanodots based LSC CDs.

**
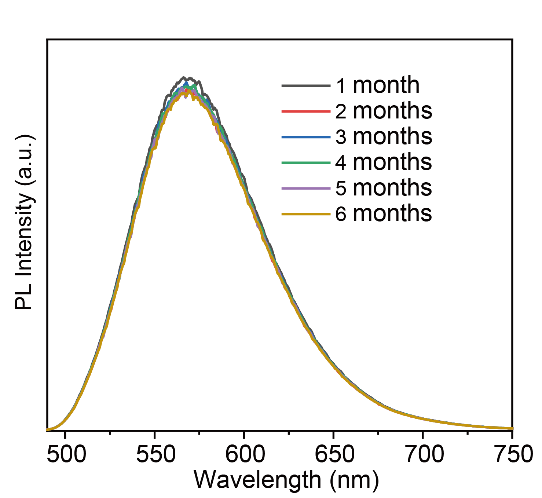
**

**Fig. S8:** PL spectra of the O-CDs after exposing to natural light for different time.

**Note S1:** With the Arrhenius curve, the value of the exciton binding energy (*E*_b_) and Huang Kun factor (*S*) for the CDs could be fitted as follows：

$I(\text{T})=\frac{I_{0}}{1+\text{A}\exp(-E_{\text{b}}/k_{\text{B}}T)}$ (1) (2)
where *I*_0_ was the emission intensity at 0 K, A was a proportional constant, and k_B_ was the Boltzmann constant, *S* was the Huang−Rhys factor and ħɷphonon was the phonon frequency.

**Analytical model for LSCs:** To calculate the theoretical external optical efficiency of LSCs, we used following formula^27^:

$\eta_{opt}=\frac{1.05\cdot(1-R)(1-e^{-<\alpha_{1}>d})\eta_{PL}\eta_{trap}}{1+\beta\alpha_{2}L(1-\eta_{PL}\eta_{trap})}$ (1)

where $\eta_{PL}$ is the PL QY of the emitters, $\eta_{trap}$ is the total internal reflection efficiency of the polymer waveguide (estimated to be around 0.75), $<\alpha_{1}>$ is the averaged absorption coefficient, $\alpha_{2}$ is absorption coefficient at the peak wavelength of the emitter, d is the thickness of the LSC, L is the length of LSC, $\beta$ is a numerical factor fixed to 1.4, R is the reflection coefficient of the air/polymer interface estimated to be 0.03. Considering the re-emitting photons, a numerical factor of 1.05 is added to correct the model. $<\alpha_{1}>$ is defined as:

$<\alpha_{1}> =-\frac{1}{d}ln(\frac{\int S_{in}(\lambda)\lambda e^{-\alpha(\lambda)d}d\lambda}{\int S_{in}(\lambda)\lambda d\lambda})$ (2)

where $S_{in}(\lambda)$ is the solar irradiance at 1.5G and $\alpha(\lambda)$ is the absorption coefficient. We used averaged value of $\alpha_{2}$ to replace the absorption coefficient and it is defined as:

$<\alpha_{2}> =\frac{\int S_{PL}(\lambda)\alpha(\lambda)d\lambda}{\int S_{PL}(\lambda)d\lambda}$ (3)

where $S_{PL}(\lambda)$ is the PL spectrum.

**Table S1.** Optical performance of QDs based LSCs comparison with reported values.

| Sample | | QY (%) | Lateral area (cm^2^) | G factor | Optical efficiency (%) | Ref | |
| --- | --- | --- | --- | --- | --- | --- | --- |
| C-dots | O-CDs | 80 | 1×5 | 5 | 5.17 | | This work |
|  |  |  | 5×5 | 50 | 0.57 | |  |
|  | Colloidal  CDs | 30 | 1.5×8 | 38 | 0.4 | | 1 |
|  | N-doped  CDs | -- | 2.5×1.6 | 4.88 | 4.75 | | 2 |
|  | Y-CDs | 92 | 5 × 5 | 6 | 3.10 | | 3 |
|  | CQDs | 65 | 15 × 15 | 4.5 | 2.2 | | 4 |
|  | CDs and nano particles | 92.2 | 5 × 5 | -- | 6.40 | | 5 |
| QDs | PbS/CdS | 40 | 1.5×10 | 10 | 6.1 | | 6 |
|  | CdSe/CdS | 45 | 1.3×21.5 | 40 | 1 | | 7 |
| Si-QDs | | 46 | 12×12 | 46 | 2.85 | | 8 |

**Reference**

1. Zhou Y, Benetti D, et al. Colloidal carbon dots based highly stable luminescent solar concentrators. Nano Energy, 2018, 44: 378-387.
2. Li Y, Miao P, et al. N-doped carbon-dots for luminescent solar concentrators. J Mater Chem A, 2017, 5: 21452-21459.
3. Guo J, Lu Y, et al. Yellow-emissive carbon dots with high solid-state photoluminescence. Adv Funct Mater, 2022, 32(20): 2110393.
4. Zhao H, Liu G, et al. Gram-scale synthesis of carbon quantum dots with a large Stokes shift for the fabrication of eco-friendly and high-efficiency luminescent solar concentrators. Energy Environ Sci, 2021, 14: 396-406.
5. Chen J, Zhao H, et al. Highly efficient tandem luminescent solar concentrators based on eco-friendly copper iodide based hybrid nanoparticles and carbon dots. Energy Environ Sci, 2022, 15(2): 799-805.
6. You Y, Tong X, et al. Eco-friendly colloidal quantum dot-based luminescent solar concentrators. Adv Sci, 2019, 6(9): 1801967.
7. Meinardi F, Colombo A, et al. Large-area luminescent solar concentrators based on ‘Stokes-shift-engineered’ nanocrystals in a mass-polymerized PMMA matrix. Nat Photonics, 2014, 8(5): 392-399.
8. Meinardi F, Ehrenberg S, et al. Highly efficient luminescent solar concentrators based on earth-abundant indirect-bandgap silicon quantum dots. Nat Photonics, 2017, 11(3): 177-185.
